# Supplementary material for: “With group antenatal care, pregnant women know they are not alone”: The process evaluation of a group antenatal care intervention in Ghana
Source: PLoS One. 2023 Nov 7;18(11):e0291855. doi: 10.1371/journal.pone.0291855 (PMC10629640; doi:10.1371/journal.pone.0291855)
Supplement: S1 Checklist — (DOCX) [file pone.0291855.s001.docx]

# S1: FIDELITY SCALE

**How to use the Fidelity Scale:** The date of the observation is placed in the top box. The form can then be used to examine fidelity to the model twice. Observers mark areas with an **x** or a **check mark** to indicate if the model is being adhered to always, sometimes, never. The *Fidelity Scale* can then be used to monitor fidelity and identify areas needing attention.

# Facilitator observed:

| **Date of Observation** |  | | |  |
| --- | --- | --- | --- | --- |
| **Fidelity Scale Items** | **Always** | **Sometime** | **Never** | **Comments** |
| 1) Small group size (no more than  14 women in a group). |  |  |  |  |
| 2) Facilitator uses learned facilitation skills |  |  |  |  |
| 1. asks empowering questions; |  |  |  |  |
| 1. encourages participation by everyone |  |  |  |  |
| 1. acknowledges comments from participants |  |  |  |  |
| 1. validates participants’ feelings |  |  |  |  |
| 1. summaries key elements of the |  |  |  |  |
| 1. listens actively |  |  |  |  |
| 3) Facilitator sits with the group and does not stand when conducting  visit. |  |  |  |  |
| 4) Follows the steps as written in  the Facilitators’ Guide. |  |  |  |  |
| 5) Displays the picture cards in a  manner so that all participants can see. |  |  |  |  |
| 6) Allows every participant an opportunity to do the demonstration/activities to reinforce the material. |  |  |  |  |
| 7) Every participant has a Take  Action Card booklet. |  |  |  |  |
