# Supplementary material for: “With group antenatal care, pregnant women know they are not alone”: The process evaluation of a group antenatal care intervention in Ghana
Source: PLoS One. 2023 Nov 7;18(11):e0291855. doi: 10.1371/journal.pone.0291855 (PMC10629640; doi:10.1371/journal.pone.0291855)
Supplement: S2 Checklist — (DOCX) [file pone.0291855.s002.docx]

**S2: LEARNING METHODS CHECKLIST**

**How to use the Learning Methods Checklist:** The date of the observation is placed in the top box. The form can then be used twice to observe group ANC meetings and see/acknowledge improvement over time with the same midwife. Observers mark each step with an **x** or a **check mark** for each step performed satisfactorily in the column under the date. The *Learning Methods Checklist* can then be used to give feedback on the meetings and identify areas for improvement.

**Facilitator observed**:

| **Date of Observation** | |  |  |  |
| --- | --- | --- | --- | --- |
| **Facilitation Skill** | | **X or check mark If skill performed correctly** | | **Comments** |
| 1) | Prepares resources and prepares for visit to start on time. |  |  |  |
| 2) | Sets up room with chairs in a circle. |  |  |  |
| 3) | Welcomes participants as they arrive. |  |  |  |
| 4) | Conducts individual exam with client either before start of group or at end. |  |  |  |
| 5) | Reviews previous meeting with participants. |  |  |  |
| 6) | Introduces today’s meeting. |  |  |  |
| 7) | Asks what the participant’s know. |  |  |  |
| 8) | Educates using large picture cards. |  |  |  |
| 9) | Walks around and shows picture card to each woman and helps locate in Take Action Card Booklets. |  |  |  |
| 10) | Shares the evidence and discusses barriers and solutions by holding up and reading back of picture card. |  |  |  |
| 11) | Facilitates discussion by reading the questions printed on the back of the picture card. |  |  |  |
| 12) | Encourages participation from women. |  |  |  |
| 13) | Comes to agreement on actions. |  |  |  |
| 14) | Interprets the picture cards (reads the back of the large picture card and waits for the women to respond) and places in a circle. |  |  |  |
| 15) | Practices the actions. |  |  |  |
| 16) | Reflects on practicing the actions. |  |  |  |
| 17) | Prepares for next meeting |  |  |  |
| 18) | Closes meeting and identifies date and time for next meeting. |  |  |  |
| 19) | Uses Facilitators Guide to read text as written during group meetings. |  |  |  |
