# Supplementary material for: “With group antenatal care, pregnant women know they are not alone”: The process evaluation of a group antenatal care intervention in Ghana
Source: PLoS One. 2023 Nov 7;18(11):e0291855. doi: 10.1371/journal.pone.0291855 (PMC10629640; doi:10.1371/journal.pone.0291855)
Supplement: S1 File — (DOCX) [file pone.0291855.s003.docx]

#

**S3 Tracking Log**

**GROUP LIST FOR TRACKING WOMEN THROUGH GROUP ANTENATAL VISITS**

| **NAME** | **PHONE** | **ALTERNATE PHONE** | **VISIT 1** | **VISIT 2** | **VISIT 3** | **VISIT 4** | **VISIT 5** | **VISIT 6** | **VISIT 7** | **VISIT**  **8** |
| --- | --- | --- | --- | --- | --- | --- | --- | --- | --- | --- |
| **1)** |  |  |  |  |  |  |  |  |  |  |
| **2)** |  |  |  |  |  |  |  |  |  |  |
| **3)** |  |  |  |  |  |  |  |  |  |  |
| **4)** |  |  |  |  |  |  |  |  |  |  |
| **5)** |  |  |  |  |  |  |  |  |  |  |
| **6)** |  |  |  |  |  |  |  |  |  |  |
| **7)** |  |  |  |  |  |  |  |  |  |  |
| **8)** |  |  |  |  |  |  |  |  |  |  |
| **9)** |  |  |  |  |  |  |  |  |  |  |
| **10)** |  |  |  |  |  |  |  |  |  |  |
| **11)** |  |  |  |  |  |  |  |  |  |  |
| **12)** |  |  |  |  |  |  |  |  |  |  |
| **13)** |  |  |  |  |  |  |  |  |  |  |
| **14)** |  |  |  |  |  |  |  |  |  |  |
