# Supplementary material for: “With group antenatal care, pregnant women know they are not alone”: The process evaluation of a group antenatal care intervention in Ghana
Source: PLoS One. 2023 Nov 7;18(11):e0291855. doi: 10.1371/journal.pone.0291855 (PMC10629640; doi:10.1371/journal.pone.0291855)
Supplement: S2 File — (DOCX) [file pone.0291855.s004.docx]

**S4 - Consolidated criteria for reporting qualitative research (COREQ)**

**Domain 1**

1. **Interviewer/facilitator:** The interviews and focus groups were facilitated by the champion trainers from the research collaborators in Ghana: Dodawa Health Research Centre.
2. **Credentials:** The research team is a combination of PhD prepared nurse-midwives, PhD/MPH, midwives, nurses, a statistician and a physician. Additionally there are research assistants from Ghana who are college graduates from various disciplines such as public health and nursing
3. **Occupation:** The researchers are either employed as faculty/staff by the University of Michigan or Dodawa Health Research Centre
4. **Gender:** The champion trainers conducting the focus groups and interviews were all female
5. **Experience and training:** The research team from University of Michigan experienced in qualitative health research provided training in facilitating focus groups and interviews as well as or the research assistants who were recording and transcribing the data.

**Relationship with participants**

1. **Relationship established:** The research team includes champion trainers who were known to the midwives from their training in group ANC.
2. **Participant knowledge of the interviewer:** The champion trainers all introduced themselves and explained the purpose of the focus groups and interviews.
3. **Interviewer characteristics:** While the interviewers we all interested and invested in the intervention, the questions asked were open-ended and included what was “not working well”.

**Domain 2: study design**

**Theoretical framework**

1. **Methodological orientation and Theory:** For the purpose of this process evaluation we used descriptive thematic analysis.

**Participant selection**

1. **Sampling:** The sample included all participants who were in the intervention arm of the cluster RCT who were present at the meeting where the focus group was conducted and who were willing to participate in the focus groups and 7 midwife facilitators
2. **Method of approach:** Participants were informed of a focus group occurring after one of the GroupANC meetings with a goal of gathering their perspective and experiences and that it was completely voluntary. The midwives were asked if they would participate in an in-person short interview to be conducted after a GroupANC meeting.
3. **Sample size:** 7 midwives were interviewed and 92 pregnant women participated in the focus groups
4. **Non-participation:** Of the women present at the GroupANC meeting, none declined to participate. All 7 midwife facilitators approached agreed to be interviewed

**Setting**

1. **Setting of data collection:** for the focus groups, data was collected at the intervention site health facility following a group ANC meeting. Interviews were conducted at the same locations with the midwives
2. **Presence of non-participants:** Only the research assistants and the participants were present at each focus group and interview.
3. **Description of sample:** Demographic data was collected for the primary research, however it was not reported for this process evaluation.
4. **Interview guide**

- Questions for semi structured focus groups
  - How did you feel about the education you received about pregnancy in group?
  - What are some of the things you liked about your meeting in groups?
  - What are you not happy about regarding the group meetings?
  - What other topics would you like to be added to your teachings?
  - Do you all have the white booklet that is used for the teachings? What benefit did you derive from the white booklet?
  - Do you have thoughts, suggestions, concerns with regards to meeting in groups?
- Questions for semi structured interviews
  - Tell me about what went well in the meeting today?
  - Tell me about what did not go well in the meeting today?
  - What is your opinion on providing antenatal care to women in groups?
  - What in your opinion was striking about the topic today?
  - What have you learnt from the meeting today? What new things have you learnt and what do you think we can add to improve on the next meeting?
  - What suggestions do you have for changing anything in today’s meeting?
  - What is your view on the facilitator’s guide you used today? Did you find the guide useful for this meeting?
  - Any other suggestions, ideas or comments about the day’s activities?
  - Did you do every activity that was scheduled for today’s meeting? Probe: If NO, which activity or activities were not done and why?
  - Are there any topics you would suggest we add to today’s meeting? If yes, what are they?
  - Are there any topics you think we should remove from today’s meeting? If yes, what are they?
  - How did the mothers receive the group antenatal concept? Do you think the mothers prefer the group antenatal care to the individual concept, explain?

1. **Repeat interviews:** there were no repeat interviews
2. **Audio/visual recording:** with permission from the participants, the focus groups and interviews were audio recorded and transcribed (the focus groups were also translated from Twi to English) by the research assistants.
3. **Field notes:** Field notes were not taken
4. **Duration:** Interviews lasted 20-30 minutes and the focus groups lasted between 30 and 40 minutes
5. **Data saturation:** Because all potential participants were included in the interviews and focus groups, saturation was not discussed
6. **Transcripts returned:** Transcripts were not returned to the participants for comments or corrections

**Domain 3: analysis and findings**

1. **Number of data coders:** Coding was conducted individually by 4 authors who then met to discuss themes
2. **Description of the coding tree:** As the qualitative data was part of a process evaluation that included other data we did not include a coding tree in the manuscript
3. **Derivation of themes:** Themes were derived from the data
4. **Software:** Transcripts were analyzed without the use of qualitative analysis software
5. **Participant checking:** for this process evaluation we did not conduct participant checking

**Reporting**

1. **Quotations presented:** Quotations were presented verbatim and were not identified by participant number
2. **Data and findings consistent:** Each author reviewed the data and findings to determine consistency
3. **Clarity of major themes:** Sub themes were identified and collapsed into major themes during analysis meetings
4. **Clarity of minor themes:** The authors met to discuss relevance of all themes to the purpose of the process evaluation and were included if they were deemed to be relevant.
5. Tong A, Sainsbury P, Craig J, Consolidated criteria for reporting qualitative research (COREQ): a 32-item checklist for interviews and focus groups, *International Journal for Quality in Health Care*, Volume 19, Issue 6, December 2007, Pages 349–357, <https://doi.org/10.1093/intqhc/mzm042>

^f^
