# Supplementary material for: “With group antenatal care, pregnant women know they are not alone”: The process evaluation of a group antenatal care intervention in Ghana
Source: PLoS One. 2023 Nov 7;18(11):e0291855. doi: 10.1371/journal.pone.0291855 (PMC10629640; doi:10.1371/journal.pone.0291855)
Supplement: Attachment — Submitted filename: null.docx [file pone.0291855.s006.docx]

**TITLE:** “With Group Antenatal Care, Pregnant Women Know They are Not Alone”: The Process Evaluation of a Randomized Controlled Trial of Group Antenatal Care in Ghana

**SHORT TITLE:** Process Evaluation of an RCT of Group Antenatal Care in Ghana

**AUTHORS**

Ruth Zielinski^1*^, Vida Kukula^2^ , Veronica Apetorgbor^2^, Elizabeth Awini^2^, Cheryl Moyer^3^, Georgina Badu-Gyan^2^, John Williams^2^, Nancy Lockhart^1^, Jody Lori^1^.

^1^ Department of Health Behavior and Biological Sciences, School of Nursing, University of Michigan, 400 N. Ingalls Bldg, Ann Arbor, MI, United States of America

^2^ Ghana Health Service, Dodowa Health Research Centre, P.O.Box DD1, Dodowa, Ghana

^3^ Department of Learning Health Sciences, University of Michigan Medical School, University of Michigan, Ann Arbor, MI, United States of America

* Corresponding author Email: ruthcnm@umich.edu

Abstract

**Background**

An essential component to improving maternal and newborn outcomes is antenatal care. Group antenatal care, where 10-14 women with similar due dates meet together for visits, includes traditional components such as risk assessment with increased opportunity for education and peer support. In this report, we describe the process evaluation of a cluster randomized controlled study of group antenatal care implemented in Ghana.

**Methods**

The process evaluation included both quantitative and qualitative data sources. Midwives at the intervention sites completed tracking logs to measure feasibility of the intervention. Research team members traveled to intervention sites where they conducted structured observations and completed fidelity and learning methods checklisst to determine adherence to the model of group antenatal care delivery. In addition, midwives facilitating group antenatal care meetings were interviews and focus groups were conducted with women participating in group antenatal care.

**Results**

In the majority of cases, midwives facilitating group antenatal care completed all components of the meetings with fidelity, following best practices such as sitting with the group rather than standing. Across 7 intervention sites, 7 groups (622 pregnant women) were documented in the tracking logs and of these participants, the majority (74%) attended more than half of the meetings, with 32% attending all 8 group meetings. Three themes were identified in both the midwife interviews and focus groups with participants: 1) information sharing, 2) sense of community, and 3) time management challenges. An additional theme emerged from the focus groups with the women: women who had already given birth noticed a disconnect between what they learned and treatment received during labor and birth.

**Conclusion**

This process evaluation determined that group antental care can be implemented with fidelity in low- and middle-income countries. Time management was the biggest challenge, however both midwives and pregnant women found the model of care not only acceptable, but preferable to traditional care. The knowledge shared and sense of community built during the meetings was a valuable addition to the individual model of antenatal care.

Introduction

Maternal and infant health have long been at the forefront of global health initiatives. Through concerted efforts to improve maternal and newborn health, some progress has been made. In 1990, newborn deaths worldwide was 5 million and by 2019 that had decreased to 2.4 million (1) During the same time period the maternal mortality ratio (MMR) (number of maternal deaths during a given time period per 100,000 live births) reduced by 38% to 211 per 100,000 live births (2). However, the MMR is still three times higher than the goal set by the United Nation’s Sustainable Development Goal (SDG) of less than 70 maternal deaths per 100,000 live births by the year 2030 (3). The newborn death rate NMR of 27/100 is more than twice as high as the goal set by the SDGs of less than 12 newborn deaths per 1000 live births by the year 2030(3). Moreover, there has not been consistent improvement across countries with 99% of maternal deaths occurring in low- and middle-income countries (LMICs) (4) . Sub-Saharan African countries alone account for 86% of maternal deaths with a MMR of 415/100,000 and NMR of 27/1000; bearing the brunt of the loss of lives among newborns and mothers (4).

Antenatal care (ANC) defined by the World Health Organization (5) as “care provided by skilled health-care professionals to pregnant women and adolescent girls to ensure the best health conditions for both mother and baby during pregnancy” (5 p. 1) is one approach to reducing negative outcomes for mothers and infants. Traditionally, ANC involves individual, brief, regular visits with a maternity care provider that focuses on risk reduction of complications such as preeclampsia (6). An innovative alternative to the traditional model of ANC is group ANC which places clinical assessment, education and social support into a group setting. These group meetings retain the risk assessment components of ANC care such as monitoring blood pressure while providing an opportunity for education, empowerment and support. In the group ANC model, women with similar estimated due dates are organized into groups that meet at regular intervals during pregnancy. Meetings typically last 1-2 hours which allows time for more in-depth discussions and participation from group members. The groups remain stable throughout, which offers the additional benefit of social support from the group.

A National Institute of Health (NIH) funded study entitled Group Antenatal Care to Promote a Healthy Pregnancy and Optimize Maternal and Newborn Outcomes: A cluster randomized controlled trial has been implemented in Ghana to determine the effectiveness of the group ANC model of care. While this manuscript focuses on the process evaluation of the study, the overarching goal is to determine whether group ANC facilitated by trained midwives, utilizing a fixed group model of 10-14 pregnant women by gestational age for the duration of the pregnancy, improves outcomes when compared with participants in routine, individual antenatal care. Details of the project have been described eslewhere (7). Briefly, the randomization occurred at the facility level whereby 7 facilities were randomized to routine ANC and 7 were randomized to offer group ANC to pregnant women seeking ANC at the facility. Primary outcomes include birth preparedness, complication readiness, increased care-seeking and improved maternal and newborn outcomes. Enrollment in the study and participating in group ANC was voluntary.

While program outcomes measure the success of an intervention, a process evaluation is important to understand why a program is, or is not, successful and to ensure that the outcomes seen are a result of the program, particularly when implementation occurs in different cultures and contexts (8). Preliminary data indicates that group ANC is feasible and may result in better outcomes for mothers and newborns in lower resourced countries (9), however larger studies of this method of ANC delivery are needed, particularly in sub-Saharan Africa given the unacceptably high rates of maternal and neonatal mortality and morbidity (5). To that end, we developed and implemented a process evaluation at the onset of the study to monitor fidelity to the intervention (table 1) and identify issues and barriers in a timely manner (10).

Table 1: Components of the GRAND - Group Antentatal Care Model

- Facilitated group ANC meetings covering the WHO recommendations on antenatal care for a positive pregnancy experience.(11)
- Groups of approximately 10 - 14 pregnant women of similar gestational age, conducted while siting in a circle.
- Groups led by two midwives trained in group facilitation and participatory learning.
- Physical assessment and procedures conducted one-on-one with midwife before or after the group ANC meetings
- Self-assessment including blood pressure, weight and whether a woman has experienced any danger signs

The purpose of this process evaluation was to identify and document patient, provider, and system barriers and facilitators to program implementation**.** Using both quantitative and qualitative methods, potential and actual influences on the quality and conduct of the program's operations, implementation, and service delivery were identified. The data collected were used for formative and summative purposes as a continuous monitoring and problem solving approach to oversee model fidelity.

Methods

**Ethics Statement**

The study was approved by the Ethical Review Committee of the Ghana Health Service and the University of Michigan Institutional Review Board.

During the summer of 2019, research team members with expertise in group ANC from the United States met with research partners in Ghana to conduct a train-the-trainer course for a selected group of midwives that would then serve as champions to train the midwives working at the intervention sites. The training was undertaken in the same manner in which the group ANC would be conducted; in small groups, in a circle, where discussion and participation were encouraged. The champion trainers (n=10) then traveled to the districts where they were supported by members of the research team as they trained an additional 52 midwives as facilitators for group ANC. Each training session lasted 3 full days and included opportunities for each midwife to practice being the facilitator. Each participant was given a Facilitator Guide and a set of facilitation tools to use to prepare for and use during each group ANC meeting.

Following the midwife facilitator training, members of the Ghana research team were given an orientation in the data collection methods for the process evaluation. The process evaluation included both qualitative and quantitative data sources (table 2) Data collection began at the implementation of the group ANC meetings and continued for the next 15 months.

Table 2: Data Sources for Process Evaluation

- Structured Observations
  - Fidelity Checklist
  - Learning Methods Checklist
- Interviews with Midwives
- Tracking Logs
- Focus Groups with Participants of Group ANC

**Structured Observations**

A sample of group ANC meetings were observed for each midwife to monitor fidelity to the model. Beginning with the initial groups in fall 2019, and continuing at intervals throughout the study period, members of the Ghana research team who had also participated as champion trainers traveled to the intervention sites to observe the facilitation of group ANC meetings. Observations included whether content was delivered as intended, whether women were encouraged to actively participate in group discussions and activities, whether picture cards were used as written in the Facilitators Guide, and whether feedback was provided to participants during demonstrations. During these observations the champion trainers also completed both the Fidelity Checklist Scale and the Learning Methods Checklist (Supplemental Materials).

The Fidelity Checklist – This scale consists of 7 items scored as “Always”, “Sometimes” or “Never”. The items identify whether or not the midwife is using the techniques taught to encourage group dynamics and discussion. For example, the midwife should sit in the group rather than stand when conducting the meeting. Additional space is available for comments at the bottom of the Fidelity Checklist.

The Learning Methods Checklist – This is a 16 item checklist that identifies whether or not the midwife follows the format spelled out in the group ANC Facilitators Guide. The format is intentionally prescriptive to ensure all topics are adequately covered and there is group discussion around the topics. For this checklist, observers were instructed to place a check mark if the item was observed. Additional space is available for comments.  **Interviews**

A face-to-face semi-structured interview was completed with each of the midwives following one of the structured observations. The purpose was to explore the midwives’ perceptions of which components of the group ANC intervention were successfully implemented, to identify barriers and facilitators to implementation and to give suggestions for improvement. The interviews were conducted in English by a member of the research team using the semi-structured interview guide (see Supplemental Materials). The interviews were audio-recorded and transcribed verbatim by research assistants.

**Tracking logs**

A brief form was completed by the midwife each time a group ANC meeting was held to track the date of the meeting and the number of participants from the group in attendance. This measured “dose” for intervention outcomes but also feasibility by determining if participants were able to attend all or most of the group ANC meetings.

**Focus Groups**

For each intervention site at least one focus group discussion was held with women participating in group ANC. These focus groups, conducted by a member of the research team, were held at the end of the group ANC meeting and were voluntary; women were welcome to leave the group if they did not want to participate. Using an open-ended question guide (see Supplemental Materials), the participants were encouraged to share their perception of group care and offer suggestions for improvement. The interviews were conducted in the local language, Twi, audio recorded and then translated and transcribed verbatim by research assistants who are fluent in both English and Twi. Key phrases that were difficult to translate were left intact, with the closest approximate meaning put in parentheses in the transcription.

**Data Analysis**

Quantitative data (Fidelity Checklist and Learning Methods Checklist) were analyzed descriptively to assess whether certain items, midwives, or intervention sites were performing less well when compared to others. Qualitative data were analyzed thematically by the research team members individually reading and re-reading the transcripts to identify key themes. The use of an audit trail composed of methodological and analytical documentation and validation with other research team members was used to achieve validity (12). Four standards for methodological congruence in qualitative research were followed including rigor in documentation, ethical rigor, procedural rigor, and auditability (12). Each data collection method was analyzed separately and then compared for congruence or dissimilarities.

Results

**Checklists**

A total of 32 Fidelty Checklist Scales were completed among the intervention sites. The majority of the items were ranked as “Always” (188/224 responses), with the remainder as “Sometimes” (36/224 responses) with none of the responses marked Never. The item that scored the lowest with the most “Sometimes” vs. “Always” was item 4 “Follows the steps as written in the Facilitators Guide” (Figure 1). Examples of comments included *“the meeting was very interactive”, “midwives should review the guide well before group meetings”* and *“ask open-ended questions to empower pregnant women to talk”*. The checklists were then reviewed with the midwife to provide timely feedback.

**Figure 1 – Fidelity Scale Scoring**

The Learning Methods Checklist with 16 items identifies which steps may have been missed in the meeting format. In most cases, observers indicated that all steps were followed, with only two observations indicating that the midwife did not “Go through the what and why” step of explaining what actions are needed when a problem arises and why those actions are taken. However some checklists included comments that reminders were given by the observers, such as to *“Review the previous meeting”.* Comments also indicated challenges in time management such as *“Meeting started an hour late”* and *“Meeting was delayed”.* Resource management was also an issue, *“BP monitors were not charged”* at one facility and another comment was *“Not all resources were ready to start meeting.”*

**Tracking Logs**

There were a total of 70 groups tracked across the 7 intervention sites between August 2019 and April 2022. A total of 622 pregnant women were documented in the tracking logs. Of these participants, the majority (74%) attended more than half of the meetings, with 32% attending all 8 group ANC meetings. There were 11% who attended only one or two meetings, typically attending the first one or two Group ANC meetings and then dropping out. This might suggest they either miscarried or moved away, yet it could indicate dissatisfaction with the group care model. However such data were not collected as most of these women were lost to follow up.

**Figure 2 – Tracking Logs**

**Interviews with Midwives**

One-to-one interviews using a semi-structured interview guide were conducted by research team members with group ANC midwives at each of the intervention sites (n=7). Three themes were identified: 1) information sharing, 2) sense of community, and 3) time management challenges.

*Information Sharing*

Midwives identified that they enjoyed sharing information with participants in a setting that offered the opportunity for group participation *“because this one is in a small group they are able to feel free and to share experiences”* and that it also allowed time to discuss the “why” versus just the “what”. *“I think the pregnant women appreciate knowing why we do something like take a blood pressure”.* Midwives indicated that having a step-by-step guide for each meeting was very useful in preparing for the meetings and in keeping the meetings running smoothly, indicating it was also a source for the midwives to gain additional knowledge: *“We also get knowledge from it.”* The midwives expressed optimism that group ANC would have long term educational benefits*. “It’s going to go a long way to help our women understand the education we give them and the care and what they are supposed to do themselves during the pregnancy, labour and after delivery”.*

*Sense of Community*

Midwives felt that group ANC was beneficial in creating community among the women where information and support was coming from other women and not just from the midwife *“Because [group ANC] is a small group they are able to feel free and to share experiences so this…is much better than before.”* Group ANC instilled trust between the woman and midwife because there was a consistent midwife in the group rather than the women seeing a different midwife each time

*Time Management Challenges*

Midwives indicated that starting on time was a challenge because not all of the women were there on time, and they needed to decide whether to finish all the material or end on time. They expressed optimism that time management would get easier with subsequent meetings: *“We are hoping that our next meeting things will be a bit faster then we minimize the number of hours or minutes we used today.”*

**Focus Groups with Participants**

A total of 10 focus group discussions with a total of 92 participants (range 5-13 per group) were conducted after the final group ANC meetings. Some women in the groups had already delivered and attended with their newborns. Focus groups lasted approximately 30 minutes. Participants were overwhelmingly positive about their participation in group ANC and hoped it would continue. Qualitative, thematic analysis of focus group data revealed that participant experiences resulted in themes identical to those in the midwifery interviews: 1) sense of community, 2) information sharing, 3) time management challenges, and 4) disconnect.

*Sense of community*

Some participants expressed initial reluctance to participate that was quickly overcome. “*On the first day it was scary…but after I got involved I realized it’s comfortable and good and it is helping me too.”* Overwhelmingly, participants expressed they were happy they joined and were proud of their participation. As one participant said after the last group ANC visit, *“Everyone has become a graduate in pregnancy”.*

Participants in each group expressed their happiness that the midwives demonstrated respectful maternity care. *“You play with us, you make us happy, and you also teach us well…we tend to forget some burdens we bring from home”.* They appreciated being treated with kindness and patience by the midwives who gave out their phone numbers, calling, and even visiting participants in their homes. *“The way the midwives treat them it shows that they have love for the pregnant woman.”*

Women came back after their babies were born to share with others. They expressed a desire to continue group ANC so other women could participate. “*When I went to deliver my first born I didn’t get this kind of treatment…I want you to continue with it so that everyone will benefit to help all women who give birth.”* Participants asked that the meetings continue through the postnatal period. Some suggested they be involved in teaching others. *“I hope that there are other pregnant women who also need the education…We could come and also teach them what we did that helped us.”*

Participants enjoyed meeting new friends and relied on others for support. *“I am happy because you have brought togetherness and love between us and that makes me happy.”* Some even suggested having a fund for those with more financial need.

*Information sharing*

Participants were able to recall all topics covered. What is evident from the data is that it was important to participants that the *why* was included. *“At first you think it is normal, but due to the teaching you will know that the sickness you have is not normal and why you need to take the medicine.”* Knowledge helped to quell fears and dispel myths. *“It has taken away the fear we used to have when pregnant”.* Participants discussed learning about the importance of preparation for birth – specifically transportation, supplies and saving money for emergencies.

Participants expressed enthusiasm for the group ANC model of care where participants check one another’s blood pressure and weights with the supervision of the midwife, expressing feelings of empowerment and agency. *“I was excited because…I check my BP and weigh myself”.* They gained confidence to ask questions of care providers. *“We were able to understand that when you come and they check you, you can ask how it is.”*

Participants in group ANC are given a booklet to take home as a helpful reminder of things they learn in the meetings. *“It’s still helping because when I see some signs and I don’t understand I open the book to check to see if it’s a danger sign or it’s normal”.* Participants used the booklet to share the information with friends and family members which helped validate the information as correct. *“Sometimes when I go and tell my husband that we learned these things, at times he doesn’t believe me but I show him the book.”* Participants recalled the topics regarding partner communication and suggested that partners be encouraged to attend some meetings. *“They taught us how we should communicate with our partners…they taught us how to talk to our partners so that they can understand and support us.”*

Women who had experienced traditional ANC in prior pregnancies indicated that group ANC was preferred. Participants expressed greater knowledge acquisition and feelings of support during group ANC. “*This will be my 5^th^ child. There are a lot of things I do not know about the previous 4 pregnancies but now…I will remember what we were taught here.”*

*Time management challenges*

Similar to the interviews with midwives, this issue was mentioned in every group. Participants shared that it was hard for them to arrive on time. *“Looking at the time scheduled for us, it is difficult for some of us to access vehicles”.* Others expressed frustration that they were there on time and had to wait. “*My problem was with the time, we come early hoping that we can go early but when we come for the meeting we stay for long.”* At times it was the midwives who did not come on time. *“Make sure they come on time and also the (midwives) should also come on time”*

*Disconnect*

At times participants who had already given birth saw a disconnect between the group ANC teaching and the birth experience. In other words, participants were empowered to advocate but on the maternity ward they were not always treated with respect. One participant recalled being discounted by the staff: “*I told them that the baby was coming, they told me that it is not yet time and that I am not the one to tell them the time to check me*.” There were stories of disrespectful maternity care: “*They kept inserting their hands inside my vagina and in fact I was not happy at all*.”

Discussion

Maternal and infant mortality in LMICs remains unacceptably high and women and infants that survive pregnancy and childbirth often suffer long term sequalae. While death rates for both mothers and newborns consistently trended downward between 2000 and 2019, there is preliminary evidence that progress has stalled or in some cases been negated by the global COVID-19 pandemic (13). This underscores the importance of implementing measures to improve maternal and newborn health that can be sustainable through events such as pandemics.

Often, the traditional approach to ANC provides little in the way of education and counseling about health-promoting behaviors. The WHO recommends a more comprehensive approach to ANC that includes routine screening, timely and comprehensive education as well as “including social, cultural, emotional and psychological support, to pregnant women in a respectful way” (4, p. ix). Providing this sort of comprehensive ANC is challenging especially in resources constrained countries. Long waiting times, lack of continuity of care by caregivers and hurried consultations due to heavy workload on health providers results in dissatisfaction and missed visits (14). To provide the most benefit, ANC must provide quality care that is respectful and valued by both the provider and the patient.

Group ANC models of care have been implemented successfully in developing countries with promising, but not consistent outcomes (6,15–18). While group ANC in Rwanda did not result in a difference in gestational age at birth or number of ANC visits attended (16), participants felt they had increased knowledge and support and a closer relationship with their providers (19). A study in Malawi found that women in the group ANC arm of the study were more likely to receive comprehensive services such as blood pressure screening at each visit and demonstrated increased knowledge of health related topics than women assigned to individual antenatal care (17). A pilot study conducted in Malawi and Tanzania found that participants in group ANC were better able to communicate with their partners and their partners were more likely to be tested for HIV (20). Preliminary research in Ghana demonstrated an increase in health literacy or more specifically, how to recognize and prevent problems, when to access care, breast feeding and family planning methods (21). Moreover, evidence from Kenya suggests group ANC may be associated with an increase in facility based births as well as family preparedness for birth (22).

Feasibility of implementing group ANC has been explored previously and our results are similar (23,24). CenteringPregnancy was adapted and implemented in Mexico and found to be feasible and acceptable to both the health care professionals and pregnant women (23). Adequate space for the group meetings and recruitment were the biggest challenges in this context (23). A generic model of group ANC was developed by researchers in India, using elements from CenteringPregnancy and Home Based Life Saving Skills (24). Feasibility and acceptability was explored qualitatively by demonstrating one meeting to key informants (maternity care providers, administrators and pregnant women). Participants were supportive of the model of care, finding it more comprehensive, educational, and empowering vs. traditional care (24).

Using a train-the-trainer model of implementation, we were able to train midwives efficiently in small groups so they could practice the facilitation techniques. They were able to administer the group ANC curricula with fidelity to the format using the techniques they learned in the training. The midwives that were interviewed all expressed a preference for group ANC over the standard, individual care model, feeling like it resulted in more education and a stronger sense of community. They appreciated the Facilitators Guide and believed it helped them to cover everything and that they, as well as the women, learned from the information. The majority of midwives followed the steps of the ANC visit according to the Facilitators Guide, however there were some components that needed to be reinforced. This included the midwife asking open ended questions to facilitate discussion as well as time management; starting and ending on time.

The research team was able to recruit pregnant participants willing to enroll in the study and participate in group ANC despite some delay due to the COVID pandemic. Of the participants, the majority attended four or more of the 8 meetings. Given that the traditional model of ANC in Ghana included 4 visits, attendance at 8 visits by more than 1/3 of the women is encouraging. Women who participated in group ANC voiced enthusiasm for the model of care and wished for it to continue. Having the opportunity to tell stories, voice opinions and ask questions in the group gave them a sense of empowerment as did self-checking blood pressures and weights. They enjoyed having materials to take home to share with friends and family. One of the most salient findings was that group ANC created a sense of community and also a connection to the midwives that they did not experience with prior antenatal care.

**Limitations**

Implementation of the group ANC intervention and the ensuing process evaluation were impacted by the pandemic, as described above. However, the research team in Ghana and the midwife facilitators were committed to the program success. Fortunately, most of the group meetings were held outside which limited risk of COVID transmission and participants and midwives were required to wear masks during the meetings. In some circumstances there was a disconnect between what the women experienced in their group ANC and at the birth facility. In Ghana, it is common for midwives to either work in the antenatal or intrapartum area and not in both..

**Conclusion**

Process evaluations are increasingly incorporated into health intervention research, providing value by documenting the barriers and facilitators of the intervention components (25)^.^ Our process evaluation was designed to monitor and document fidelity to the core components of the group ANC intervention. As noted, challenges were faced in time management, yet overall acceptability by midwives and women as well as fidelity to the group ANC model was high.

Significant shifts in clinical practice do not happen easily or quickly. Valid, reproducible evidence must exist for practice guidelines to change. Our findings contribute to the growing evidence that group ANC in LMICs is safe, feasible and that women find it beneficial. We advocate for future research focusing on the scale-up of group ANC in LMICs to address the unacceptable high rates of maternal and newborn mortality.

References

1.	WHO. Newborns: improving survival and well-being. World Heal Organ [Internet]. 2020;(Mlcc):1–5. Available from: https://www.who.int/news-room/fact-sheets/detail/newborns-reducing-mortality

2.	WHO | Trends in maternal mortality: 1990 to 2015. WHO [Internet]. 2016 [cited 2018 Feb 19]; Available from: http://www.who.int/reproductivehealth/publications/monitoring/maternal-mortality-2015/en/

3.	United Nations. Sustainable Development Goals Available from: https://sdgs.un.org/goals

4.	Graham W, Woodd S, Byass P, Filippi V, Gon G, Virgo S, et al. Diversity and divergence: the dynamic burden of poor maternal health. Vol. 388, The Lancet. 2016. p. 2164–75.

5.	WHO recommendations on antenatal care for a positive pregnancy experience [Internet]. [cited 2022 May 17]. Available from: https://www.who.int/publications/i/item/9789241549912

6.	McDonald SD, Sword W, Eryuzlu LE, Biringer AB. A qualitative descriptive study of the group prenatal care experience: Perceptions of women with low-risk pregnancies and their midwives. BMC Pregnancy Childbirth. 2014;14(1):1–12.

7.	Williams J, Kukula V, Apetorgbor V, Awini E, Lockhart N, James K, et al. GRoup ANtenatal care Delivery (GRAND): study protocol for a randomized trial to promote a healthy pregnancy and improve maternal and newborn outcomes. Under Rev.

8.	Steckler AB, Linnan L IB. Process evaluation for public health interventions and research. San Francisco: Jossey-Bass; 2002.

9.	Sharma J, O’Connor M, Rima Jolivet R. Group antenatal care models in low- and middle-income countries: a systematic evidence synthesis. Reprod Health [Internet]. 2018 Mar 5 [cited 2022 Jan 17];15(1). Available from: /pmc/articles/PMC5836451/

10.	Saunders RP, Evans MH, Joshi P. Developing a Process-Evaluation Plan for Assessing Health Promotion Program Implementation: A How-To Guide. Health Promot Pract. 2005;6(2):134–47.

11.	World Health Organization. Intrapartum care for a positive childbirth experience WHO recommendations [Internet]. 2018 [cited 2020 Aug 1]. Available from: http://apps.who.int/bookorders.

12.	Burns N. Standards for Qualitative Research. Nurs Sci Q. 1989;2(1):44–52.

13.	Chmielewska B, Barratt I, Townsend R, Kalafat E, van der Meulen J, Gurol-Urganci I, et al. Effects of the COVID-19 pandemic on maternal and perinatal outcomes: a systematic review and meta-analysis. Lancet Glob Heal [Internet]. 2021 Jun 1 [cited 2022 May 24];9(6):e759–72. Available from: http://www.thelancet.com/article/S2214109X21000796/fulltext

14.	Teate A, Leap N, Rising SS, Homer CSE. Teate, A, Leap, N, Rising S, Homer CSE. (2011) Women’s experiences of group antenatal care in Australia – The CenteringPregnancy Pilot Study. Midwifery. 2011;27(2):138–45.

15.	Sharma J, O’Connor M, Rima Jolivet R. Group antenatal care models in low- and middle-income countries: A systematic evidence synthesis. Reprod Health. 2018 Mar 5;15(1).

16.	Sayinzoga F, Lundeen T, Musange SF, Butrick E, Nzeyimana D, Murindahabi N, et al. Assessing the impact of group antenatal care on gestational length in Rwanda: A cluster-randomized trial. PLoS One [Internet]. 2021 Feb 1 [cited 2022 Jan 17];16(2). Available from: /pmc/articles/PMC7853466/

17.	Liese KL, Kapito E, Chirwa E, Liu L, Mei X, Norr KF, et al. Impact of group prenatal care on key prenatal services and educational topics in Malawi and Tanzania. Int J Gynaecol Obstet [Internet]. 2021 Apr 1 [cited 2022 Jan 17];153(1):154. Available from: /pmc/articles/PMC8073885/

18.	Lori JR, Ofosu-Darkwah H, Boyd CJ, Banerjee T, Adanu RMK. Improving health literacy through group antenatal care: a prospective cohort study. BMC Pregnancy Childbirth [Internet]. 2017 Jul 14 [cited 2022 Apr 8];17(1). Available from: https://pubmed.ncbi.nlm.nih.gov/28705179/

19.	Musabyimana A, Lundeen T, Butrick E, Sayinzoga F, Rwabufigiri BN, Walker D, et al. Before and after implementation of group antenatal care in Rwanda: a qualitative study of women’s experiences. Reprod Health [Internet]. 2019 Jun 27 [cited 2022 Jan 17];16(1). Available from: /pmc/articles/PMC6595554/

20.	Jeremiah RD, Patel DR, Chirwa E, Kapito E, Mei X, McCreary LL, et al. A randomized group antenatal care pilot showed increased partner communication and partner HIV testing during pregnancy in Malawi and Tanzania. BMC Pregnancy Childbirth [Internet]. 2021 Dec 1 [cited 2022 Jan 17];21(1). Available from: https://pubmed.ncbi.nlm.nih.gov/34819018/

21.	Lori JR, Ofosu-Darkwah H, Boyd CJ, Banerjee T, Adanu RMK. Improving health literacy through group antenatal care: a prospective cohort study. BMC Pregnancy Childbirth [Internet]. 2017 Jul 14 [cited 2022 Jan 17];17(1). Available from: /pmc/articles/PMC5513199/

22.	Id LG, Suhowatsky S, Kabue MM, Noguchi LM, Mohan D, Karnad SR, et al. Impact of group antenatal care ( G-ANC ) versus individual antenatal care ( ANC ) on quality of care , ANC attendance and facility- based delivery : A pragmatic cluster- randomized controlled trial in Kenya and. PLoS One. 2019;14(10):1–18.

23.	Heredia-Pi IB, Fuentes-Rivera E, Andrade-Romo Z, Bravo Bolaños Cacho M de L, Alcalde-Rabanal J, Jurkiewicz L, et al. The Mexican Experience Adapting CenteringPregnancy: Lessons Learned in a Publicly Funded Health Care System Serving Vulnerable Women. J Midwifery Womens Health [Internet]. 2018 Sep 1 [cited 2022 Apr 8];63(5):602–10. Available from: https://pubmed.ncbi.nlm.nih.gov/30199143/

24.	Rima Jolivet RR, Uttekar BV, O’Connor M, Lakhwani K, Sharma J, Wegner MN. Exploring perceptions of group antenatal Care in Urban India: Results of a feasibility study. Reprod Health. 2018 Apr 3;15(1).

25.	Schneider M, Hall WJ, Hernandez AE, Hindes K, Montez G, Pham T, et al. Rationale, design and methods for process evaluation in the HEALTHY study. Int J Obes (Lond) [Internet]. 2009 Aug [cited 2022 May 24];33 Suppl 4(Suppl 4). Available from: https://pubmed.ncbi.nlm.nih.gov/19623191/

**Supporting Information**

**S1 Fidelity Checklist**

**S2 Learning Methods Checklist**

**S3 Tracking Log**
