# Supplementary material for: “With group antenatal care, pregnant women know they are not alone”: The process evaluation of a group antenatal care intervention in Ghana
Source: PLoS One. 2023 Nov 7;18(11):e0291855. doi: 10.1371/journal.pone.0291855 (PMC10629640; doi:10.1371/journal.pone.0291855)
Supplement: Attachment — Submitted filename: Response to reviewers.docx [file pone.0291855.s007.docx]

REVISIONS RESPONSE TABLE – “With group antenatal care, pregnant women now they are not alone”: The process evaluation of a randomized controlled trial of group antenatal care in Ghana

TITLE REVISED TO: “With group antenatal care, pregnant women know they are not alone”: The process evaluation of a group antenatal care intervention in Ghana

| Editors Comments | response |
| --- | --- |
| Please ensure that your manuscript meets PLOS ONE's style requirements, including those for file naming.  https://journals.plos.org/plosone/s/file?id=wjVg/PLOSOne_formatting_sample_main_body.pdf  <https://journals.plos.org/plosone/s/file?id=ba62/PLOSOne_formatting_sample_title_authors_affiliations.pdf> | Thank you, the manuscript has been edited and revised to meet these requirements. |
| Please include a complete copy of PLOS’ questionnaire on inclusivity in global research in your revised manuscript. Please upload a completed version of your questionnaire as Supporting Information when you resubmit your manuscript. | Completed and uploaded as supporting information – thank you |
| 3. Please provide additional details regarding participant consent. In the ethics statement in the Methods and online submission information, please ensure that you have specified what type you obtained (for instance, written or verbal, and if verbal, how it was documented and witnessed). If your study included minors, state whether you obtained consent from parents or guardians. If the need for consent was waived by the ethics committee, please include this information. | Additional details have been included (page 7 line 125-133) |
| Once you have amended this/these statement(s) in the Methods section of the manuscript, please add the same text to the “Ethics Statement” field of the submission form (via “Edit Submission”). | The same text was added to the “Ethics Statement” field of the submission |
| We note that you refer to a Clinical Trial (.........) in your manuscript. Please include the name of the registry and the registration number (e.g. ISRCTN or ClinicalTrials.gov) in your manuscript. If the results have been previously reported, please provide a reference to the publication. | Clinical trial # NCT 04033003 added on page 6 line 124 of the manuscript. |
| . Thank you for stating the following financial disclosure: " Author JL received the award National Institute of Health/National Institute of Nursing Research https://www.ninr.nih.gov/The funders had no role in study design, data collection and analysis, decision to publish, or preparation of the manuscript"  At this time, please address the following queries:  a)   Please clarify the sources of funding (financial or material support) for your study. List the grants or organizations that supported your study, including funding received from your institution.  b)    State what role the funders took in the study. If the funders had no role in your study, please state: “The funders had no role in study design, data collection and analysis, decision to publish, or preparation of the manuscript.”  c)   If any authors received a salary from any of your funders, please state which authors and which funders.  d)  If you did not receive any funding for this study, please state: “The authors received no specific funding for this work.”  Please include your amended statements within your cover letter; we will change the online submission form on your behalf. | Thank you, our response has been included in the updated cover letter |
| We note that you have stated that you will provide repository information for your data at acceptance. Should your manuscript be accepted for publication, we will hold it until you provide the relevant accession numbers or DOIs necessary to access your data. If you wish to make changes to your Data Availability statement, please describe these changes in your cover letter and we will update your Data Availability statement to reflect the information you provide. | All repository data is compiled and will be uploaded to Deep Blue on acceptance of the manuscript, thank you. |
| Please ensure that you refer to Figure 2 in your text as, if accepted, production will need this reference to link the reader to the figure. | Reference was made to Figure 2 (page 12 line 238) |
| Please include captions for your Supporting Information files at the end of your manuscript, and update any in-text citations to match accordingly. Please see our Supporting Information guidelines for more information: http://journals.plos.org/plosone/s/supporting-information. | The manuscript has been edited to include captions and in-text citations using the guidelines supplied, thank you. |
| Page 2, line 27: Interviews-Please change to interviewed Page 3, lines 62: Antenatal care (ANC) defined by the World Health Organization (5) Comment: Consider removing the reference (5) since this is still reflected at the end of the whole statement. Page 3, line 64: Please delete P.1 in the reference in bracket. | Interviews changed to interviewed  (5) removed  P.1 deleted |
| Page 4, line 82: Details of the project have been described elsewhere (7). The study noted that details of the project have been described elsewhere. However, the quoted reference cannot be accessed thereby making review of the detailed project protocol impossible. This reference should be properly cited. Additionally, it is not completely in order that a funded cluster randomized trail protocol was not registered or published before commencement of the study. A more detailed description of what was done should be included in this manuscript in the absence of a link for readers to assess the detailed protocol. This will enable easy understanding of the entire project from which the process evaluation is being conducted. | I apologize that this was not clear. The protocol has since been published and the reference has been updated (page 5 line 100). The protocol was registered and available at https://clinicaltrials.gov/ct2/show/NCT04033003?term=04033003&draw=2&rank=1 |
| It may be worthwhile to explain the theoretical framework(s) considered for the conduct of the trial. This will help readers to interpret the process evaluation findings | Reference has been made to the Protocol Paper text was added to this paper (p5 l99-100) |
| This process evaluation did not mention or consider the other arm of the cluster randomized study. It is therefore not clear how the interpretation of findings at the end of the study would be done if this important arm was completely not mentioned in the process evaluation. This is a major flaw in the design of the process evaluation. | I apologize that the way it was written in the last draft was confusing. This was a process evaluation of the intervention (group antenatal care) rather than of the entire randomized study. We have added clarification of this throughout the paper. Thank you for helping us add clarity. To this end we also revised the title from “The process evaluation of a Group Antenatal Care Intervention” vs. Process evaluation of an RCT…” |
| Page 10, lines 201-205 Comment: One of the essences of process evaluation is to see whether the project is on track to deliver on intended goals as well as shape modifiable factors to help achieve the expected outcomes. It would have been appropriate to include opportunities to track this group of women as their non-attendance could be from dissatisfaction with how the project is being implemented. Their loss has closed an opportunity to learn and improve thereby diminishing the benefits of the process evaluation. | This is definitely a challenge, attempts were made to follow up with women when they did not attend but we do not have data as to reasons for discontinuation. We agree, this would have been valuable information to have and include and have added this to the limitations of the study (page 20 line 424) |
| Page 12, lines 254-257: Comment: If truly the participants were called and visited at home, the 11% lost to follow up should have been identified and interviewed on the possible reasons for their non-continuity with the ANC rather than speculating the reasons in the discussion section. | That we did not collect reasons which was a limitation of the study. A point of clarification is that it was not part of the protocol call and/or visit, this was an observation made by the participants. |
| Page 14, line 293: Time management challenges Comment: This barrier or challenge should be discussed in the discussion section on how to improve this before the end of the project | Thank you for this suggestion, a section was added to the discussion “Facilitators and barriers (page 18 line 377) |
| Page 15, line 303. Comment: It will be appropriate to consider expanding on this in the discussion section. For example, will there be need to consider including recent advancements and information during the ANC sessions so that even those who have delivered before will look forward to learning something new? | Interesting point, those who had given birth before expressed their preference for group ANC which highlights the value of not only the education but also community. Additional information added (page 20 line 433-435) |
| Additional comment on the result: The qualitative component should please be reported in accordance with the COREQ guideline with attachment of same as a supplementary material. | Additional text regarding the qualitative component was added to the manuscript and a 32 item COREQ checklist was added to the supplementary material . |
| Page 15, lines 310-324 These appeared like repetitions of what had been said in the background section. Preferably a statement or two on the essence of process evaluation and followed by the discussion of findings would suffice. | Thank you for this observation, this content has been revised with some that was not repetition of the introduction moved there and the repetitious content was deleted. |
| Lessons learnt particularly the challenges and obstacles to implementation have not been discussed. Hence losing a component of the essence of process evaluation. | Thank you for this observation – a sub-section titled “Facilitators and barriers” has been added |
| The result and discussion should be revised to include the control arm of the study which an important 50% of the study design. | I apologize for our lack of clarity that led to confusion on the part of all reviewers. This was a process evaluation of the implementation of the intervention not both arms of the study – We have made changes throughout the manuscript |
| The results and discussion section should include the control arm of the cluster randomized trial | This was a process evaluation of the implementation of the intervention not both arms of the study – We have made changes throughout the manuscript and appreciate the confusion this caused |
| The reference of the study protocol being considered for review should be properly cited. However, if the link is not available for readers to assess, authors should include a brief description of what was done in the groups | This has been updated to include the reference which is now published and available – thank you. |
| The discussion section should highlight implications of the barriers and challenges to uptake of the group antenatal care and possible ways of addressing these barriers towards attaining the project goals. | Thank you for this suggestion. A sub-section titled “Facilitators and Barriers” has been added. |
| Reviewer 1 |  |
| the clients are randomized into two groups (one received routine ANC and the other group ANC).Authors should provide the findings from the routine ANC group (primary outcomes) in the result section. | I apologize for our lack of clarity that led to confusion on the part of all reviewers. This was a process evaluation of the implementation of the intervention not both arms of the study – We have made changes throughout the manuscript |
| The discussion should be based on the availability of the data above. | Apologies - See above |
| Authors should use statistical analysis to compare the two models of ANC to make inference | Apologies - See above |
| Reviewer 2 |  |
| Title: Line 1: ‘Are’ should be capitalized | Thank you for noticing this – the title has been edited to correct PLOS-ONE format |
| Title: Line 1: ‘Are’ should be capitalized Abstract Lines 25/27/39: correct typographical errors. Authors may consider hiring a copyeditor. | Thank you for your attention to these details, the corrections have been made. |
| Line 64: clarify reference Line 82: First sentence of line 82 may come at the end of the paragraph, while line 82-87 should be moved to line 78. Line 82: correct spelling of ‘elsewhere’ | The corrections have been made, thank you |
| Line 85: qualify care seeking eg care seeking behaviour | Edited to care seeking behavior, thank you |
| Line 95: may qualify study eg GRAND study | Edited to GRAND study, thank you |
| Line 99 to 103: should be paraphrased to reflect the aim of the study not results. | This was edited to reflet the aim (p 6 114-119) |
| It is suggested to add subheadings and write them in order for example, Study setting and design, sample size, research team/data collection, data analysis (more details), ethics statement (approval no. should be included) | Thank you for this suggestion, subheadings were added, however they did not fit as well into the traditional sub-headings given that this was a process evaluation. |
| Line 108-117 can be under study setting and design, however, more context should be added. | These edits were made, thank you |
| Line 118-119: it is unclear who the process evaluators were and if they were separate from the outcome evaluators. If same, how do the authors ensure the results are not biased. | The champion trainers (who also conducted the training) conducted the process evaluation. The outcome evaluation was collected by the Research assistants. |
| Line 118-165 should be under data collection and should be further elucidated | This content was moved to data collection and some additional clarification was added |
| Line 121: Table 2 should not cross a page | Corrected, thank you |
| Line 154: It is suggested the word ‘dose’ be defined in the introduction section as well as ‘fidelity’, being the key components of the process evaluation. | Thank you for this suggestion, for clarity, rather than placing the definitions in the introduction, “dose” and “fidelity” were defined at the first point in which they were introduced in the manuscript. |
| A brief demographics of the participants (both midwives and women) included in the study may be provided | None for this group of participants |
| Line 178: correct typographical error Line 180: the word ‘Never’ should be in parenthesis Line 180-182: this is not what is reflected in Figure 1 Figure 1: ‘Never’ was on the scale and ‘Missing’ on the figure, there should be consistency. Proper title and axes labels should be added. Line 182: it is unclear who made the comments, you may wish to specify Line 182: ‘midwives’ rather than ‘midwife’ Figure 2: Proper title and axes labels should be added. Line 226: Paraphrase eg a sense of community or community partnership Line 240: indicate the number of intervention sites the focus groups were held Line 256: is this statement made by a pregnant woman? If yes, why the use of the word “them” Line 313: cross-check Line 320: clarify reference | Typographical errors were corrected, thank you.  “Never” was placed in quotations  Thank you for finding the discrepancy between the text and Figure 1. This was corrected in the manuscript. As there were no “never” responses it was not included on the figure  Thank you for bringing this lack of clarity to our attention. We clarified in the manuscript that the comments were added on the Fidelity Checklist sheets by the champion trainers during their observation of the group ANC meetings.  “community” was edited to “a sense of community”  All intervention sites had at least 1 focus group and 2 were conducted at 3 of the sites. This information has been added to the manuscript.  Line 313 – this content was moved to the introduction and seems to be consistent across all articles I could find that the pandemic has resulted in worsening outcomes for mothers and neonates.  We recognize that the use of “them” is confusing, it was how it was translated from Twi to English. We added “us” in parenthesis for clarity.  Line 320 – this content was removed from the manuscript in response to an earlier reviewer suggestion |
| It is unclear if the discussion was in line with the aim of process evaluation. More context should be given about process evaluation including the data collection tools while relating the information to the GRAND study. | The discussion section has undergone revision with these suggestions in mind |
| Barriers and facilitators to the implementation of group antenatal care identified from this process evaluation should be elucidated. | A sub-section titled “facilitators and barriers” has been added |
| The answers to the questions listed in lines 128-131 should be discussed to describe the fidelity of the model while the results of the tracking logs should be discussed to describe the dose of the model. | This has been incorporated into the discussion section |
| Line 379-383 may be moved to the discussion section. | This content was moved to the discussion section |
| Please give a concise conclusion. | The conclusion was edited for conciseness. |
