# Supplementary material for: “With group antenatal care, pregnant women know they are not alone”: The process evaluation of a group antenatal care intervention in Ghana
Source: PLoS One. 2023 Nov 7;18(11):e0291855. doi: 10.1371/journal.pone.0291855 (PMC10629640; doi:10.1371/journal.pone.0291855)
Supplement: Attachment — Submitted filename: Author responses.docx [file pone.0291855.s008.docx]

| Suggested revision | Author’s response |
| --- | --- |
| Clearly articulate and describe the study population and settings, sample size calculation, how the sample size for each study components were derived, and the data collection procedure for these components | Thank you for this suggestion. We added content to better describe the study participants, setting and data collection procedures. Since this is a process evaluation, sample size calculations were not included in this report. The study protocol is referenced in the text. |
| The authors claimed they conducted both in-depth interviews (IDIs) and focus group discussions (FGDs) but did not clearly define the processes they followed in conducting these interviews. It is unclear how the data was collected and analyzed. For instance, how many IDIs and FGDs were conducted; how many participants were included in each FGDs. Also, they need to state what measures were used to determine the inclusion criteria for participating in the IDIs and FDGs. More so, the demographic characteristics of the participants should be include. | More information has been added regarding how the interviews and focus groups were conducted. Inclusion criteria has been added. Regarding demographic characteristics. This data was collected for the group ANC outcome evaluation and will be included in those reports, however for the process evaluation the focus group data were analyzed and presented without using any identifiers such as demographic data or facility name. We did not collect demographic data for the midwife facilitator participants. |
| It is unclear the way these sections currently reads. For instance lines 237-243 (page 12 of the manuscript) include useful information that will help readers understand the study population and study setting, yet not described in the methods. It is unclear what these intervention sites are. | Additional content has been added that describes the study population, setting and intervention sites. |
| For easy of understanding and replication of the study, I will suggest the methods section follow this order: Study design, study setting, study population, sampling, data collection process, data analysis and ethical approval. See example below: | The methods section has been modified to follow the order suggested |
| The qualitative analysis plan is vague. It is unclear what types of analyses was performed for both the IDIs and FGDs. For instance, the authors mentioned in lines 207-213 (page 10-11), that “Qualitative data were analyzed thematically…”, but failed to describe in detail how the data from the participants were analyzed. Was this done inductively and deductively or both? These are very useful details and yet not discussed in depth. I would like to see a clear analysis plan in the methods. This should clearly be stated for easy replication of the study. | We agree that the qualitative analysis was not well described in the manuscript. We used a qualitative comparison design but did not identify that in the manuscript. We added the steps taken in the analysis and added a reference for qualitative comparison designs. |
| I found it very difficult to understand and follow the results section due to the style adopted. The way the qualitative data is presented makes it difficult to read. All quotes should be referenced using codes to protect the identity of the participants. I will suggest using codes such as the ones used in Pilgrim N, Jani N, Mathur S, Kahabuka C, Saria V, Makyao N, et al. Provider perspectives on PrEP for adolescent girls and young women in Tanzania: The role of provider biases and quality of care. PloS one. 2018; 13(4):e0196280. <https://doi.org/10.1371/journal.pone.0196280> PMID: 29702659. | As this was the report of a process evaluation, we did not collect identifiers for the participants and thus did not need to use codes to protect the identity of the participants. Regarding the presentation of the qualitative data, we used a presentation style similar to a process evaluation published in Plos One in 2021 that seems a better fit for this manuscript as it is also a process evaluation: Arthur K, Christofides N, Nelson G. Process evaluation of a pre-adolescent transdisciplinary health intervention for inter-generational outcomes. PLoS One. 2021 Dec 23;16(12):e0261632. doi: 10.1371/journal.pone.0261632. PMID: 34941911; PMCID: PMC8699635. |
